# The impact of age‐relevant and generic infographics on knowledge, attitudes and intention to attend cervical screening: A randomized controlled trial

**DOI:** 10.1111/bjhp.12695

**Published:** 2023-09-28

**Authors:** Frances Waite, Laura A. V. Marlow, Martin Nemec, Jo Waller

**Affiliations:** ^1^ Cancer Prevention Group, School of Cancer & Pharmaceutical Sciences King's College London London UK; ^2^ Behavioural Science and Health, Institute of Epidemiology & Health University College London London UK

**Keywords:** cervical cancer, infographic, intervention, RCT, screening intention, targeting

## Abstract

**Objectives:**

Cervical screening uptake in England is falling. Infographics could strengthen intention to attend, increase positive attitudes and improve knowledge. Age targeting could improve these outcomes further. We tested the impact of generic and age‐targeted infographics.

**Design:**

A randomized controlled trial using an age‐stratified, parallel‐group design.

**Methods:**

Women aged 25–64 (*n* = 2095) were recruited through an online panel and randomized to see one of the three infographics. We tested: (i) impact of a generic cervical screening infographic compared to a control infographic on an unrelated topic with all screening age women and (ii) impact of an age‐targeted infographic compared to a generic cervical screening infographic with older women (50–64 years). Intentions, knowledge and attitudes were measured.

**Results:**

Women aged 25–64 years who viewed the generic infographic had significantly higher intentions [*F*(1, 1513) = 6.14, *p* = .013, $\eta_{p}^{2}$ = .004], more accurate beliefs about the timeline of cervical cancer development (OR: 5.18, 95% CI: 3.86–6.95), more accurate social norms (OR: 3.03, 95% CI: 2.38–3.87) and more positive beliefs about screening benefits (OR: 2.23, 95% CI: 1.52–3.28) than those viewing the control infographic. In the older age group, there was no significant difference in intention between those viewing the generic versus age‐targeted versions [*F*(1, 607) = .03, *p* = .853, $\eta_{p}^{2}$ < .001], but the age‐targeted version was more engaging [*F*(1, 608) = 9.41, *p* = .002, $\eta_{p}^{2}$ = .015].

**Conclusions:**

A cervical screening infographic can result in more positive attitudes and better knowledge and may have a small impact on intentions. Although age targeting did not affect intention, it had a positive impact on engagement and may therefore be useful in encouraging women to read and process materials.

## INTRODUCTION

Over 30% of women aged 25–49 years and 25% of women aged 50–64 years in England have not attended cervical screening within the recommended 3‐ or 5‐year interval respectively (NHS Digital, 2022). This is concerning as not attending screening increases the risk of cervical cancer incidence and mortality (Landy et al., 2016). It is estimated that if everyone eligible in England regularly attended screening, the proportion of cervical cancer deaths prevented due to the screening programme would increase from 70% to 83% (Landy et al., 2016). Key barriers to attending cervical screening include embarrassment, fear of pain, logistics of attending an appointment and lack of knowledge (Bukowska‐Durawa & Luszczynska, 2014; Chorley et al., 2017; Wilding et al., 2020). Interventions to address these barriers could include changing the procedure (e.g., offering self‐sampling), changing opportunities (e.g., offering more appointment times or different ways of booking, sending reminders) or changing beliefs and attitudes about screening among those invited (with messaging in information materials or campaigns).

In a Cochrane review evaluating interventions to increase cervical screening uptake, invitation letters were found to be the most effective strategy (Staley et al., 2021). There was also evidence that educational materials can increase uptake, with a meta‐analysis of eight trials finding that printed educational materials increased uptake compared to a control group who did not receive this information (RR: 1.23, 95% CI: 1.05–1.44; Staley et al., 2021). Generally, it seems the effectiveness of educational interventions designed to increase cervical screening uptake and encourage informed choice increases with intensity. Previous research suggests that sending detailed information booklets alongside invitation letters does not significantly improve uptake compared to letters alone (Acera et al., 2017; Eaker et al., 2004; Radde et al., 2016). A study using photo‐comics and radio‐dramas to portray cervical cancer narratives concluded that only the radio‐drama was likely to be effective (Risi et al., 2004). Outreach programmes, which include a range of interactive components such as videos, games and activities targeting barriers and facilitators to uptake (Byrd et al., 2013) and education sessions utilizing behaviour change techniques such as cues to action and goal setting (Fang et al., 2007) can also improve uptake, but are more intensive than printed interventions and come with significant cost implications.

One format for presenting printed educational materials is an infographic: a visual representation of information, often including pictures, text and data. There has been limited research looking at infographics in the context of cervical screening uptake. However, studies have shown that infographics can help to improve understanding of, and engagement with, health information such as probability messages (Hawley et al., 2008; McCrorie et al., 2016; Spiegelhalter et al., 2011). In the context of the NHS Cervical Screening Programme, an infographic would be a simple, low‐cost intervention that could be included alongside invitation letters. These are currently posted to women, but an infographic could also be shared in a digital format if there is a shift to electronic invitations.

Infographics could also be targeted. Targeting is an appropriate alternative to tailored information when individual assessment is not feasible (Schmid et al., 2008). It encourages a focus on the similarities in characteristics and motivations of a group to address barriers to behaviour change. At present individual assessment is not feasible in a national screening programme, but targeting information by age is a possibility. Targeted information is more likely to be actively and carefully processed compared to generic information (Hawkins et al., 2008; Kreuter & Wray, 2003).

We developed a generic screening infographic (i.e., without age‐specific information) and an age‐targeted version for women aged 50–64 years. With an increasing proportion of younger women, especially those under 30, protected from cervical cancer through the HPV vaccination programme (Falcaro et al., 2021) and the highest rates of cervical cancer expected to shift from late 20s to late 50s by 2036–2040 (Castanon et al., 2018), we felt targeting women in the older cohort was important. Screening is the only prevention method available for older women and evidence suggests that regular attendance between the ages of 50 and 64 years can continue to offer a woman protection against cervical cancer through her 60s and into her 70s and 80s (Castañón et al., 2014). In addition, evidence suggests that older women are more likely to be active decliners, in part, because they no longer feel at risk of cervical cancer and perceive fewer benefits to attending (Marlow et al., 2018). An information‐based intervention could therefore be more effective in the older age group. The infographics contained five theory‐informed messages developed in a previous study (Marlow et al., 2021). The messages aimed to target reflective motivation and psychological capability using education (to improve knowledge and understanding) and persuasion (inducing positive or negative feelings) in line with the COM‐B framework (Michie et al., 2011). In previous research it was found that individual messages did not influence intention strength, but seeing multiple messages did (Marlow et al., 2021; Waite et al., 2022).

The aims of this study were (i) to test the impact of a cervical screening infographic for all screening age women on intention, knowledge and attitudes and (ii) to test the impact of an age‐targeted infographic for older women on intention, knowledge, attitudes and engagement. Our hypotheses were:

1. Women aged 25–64 years who read a cervical screening infographic will have higher intentions to attend cervical screening than women who read a control infographic.
2. Women aged 25–64 years who read a cervical screening infographic will have better screening related knowledge and more positive attitudes towards screening than women who read a control infographic.
3. Women aged 50–64 years who read an infographic targeted to their age group will have higher intentions to attend cervical screening than women who read a generic cervical screening infographic.
4. Women aged 50–64 years who read an infographic targeted to their age group will have better screening related knowledge and more positive attitudes to screening than women who read a generic cervical screening infographic.
5. Women aged 50–64 years who read an infographic targeted to their age group will find it more engaging than a generic cervical screening infographic.

## METHODS

### Design

An online survey using an age‐stratified (ages 25–49 years and 50–64 years), parallel‐group randomized controlled trial design. Data were collected over 3 weeks in August 2022 until we had reached the pre‐determined sample size (details below). The protocol and questionnaire are available on Open Science Framework (OSF; https://osf.io/6fbrx). The CONSORT checklist is provided in File S1. A pilot study was conducted in June and July 2022 using a convenience sample of *N* = 932 women aged 25–64 years living in England and was publicized by the Women's Institute. The study helped to inform sample size estimations for the present study. The results of the pilot study are available in a report on OSF (https://osf.io/6yhwz). The study was given ethical approval by a UK university (LRM‐21/22‐27098) as an amendment to our pilot study on 8 July 2022.

### Participants

Participants were recruited through online panels managed by the market research company Savanta. Women were emailed or sent a notification via an app and provided with a link to the survey, hosted by the research team on SurveyMonkey. The topic of the study was not included in the initial email to avoid response bias. Participants were paid between 50p and £2 (the incentive increased towards the end of recruitment to encourage participation). Participants were eligible if they were aged 25–64 years, lived in England, had no history of cervical cancer, had access to a computer, laptop or tablet to complete the survey and met the quotas (details below) for age, ethnicity and education.

### Procedure

After consenting, women answered eligibility and demographic questions relating to the quotas. Women who met the inclusion criteria completed baseline measures. Participants were then randomized to see an infographic. No limits were imposed on the time participants spent looking at the infographic, but they were not able to view it while completing the post‐exposure measures. After exposure to the infographic, participants completed a set of outcome measures. Participants were also asked a single‐item attention check question.

### Exposure

Women aged 25–49 years were randomized (1:1) to view either: (i) a generic infographic on cervical screening or (ii) a ‘control’ infographic. Women aged 50–64 were randomized (1:1:1) to view either: (i) an age‐targeted infographic on cervical screening, (ii) a generic infographic on cervical screening or (iii) a ‘control’ infographic. Randomization was on an individual basis using SurveyMonkey's page randomization option.

Two infographics presenting information related to cervical screening were created in partnership with behaviour change communication agency Claremont. Key messages used in the screening infographics are presented in Table 1. We gave Claremont the messages to include, but the infographic was developed by the agency with input from four co‐design sessions with women aged 50–64 years from a range of socio‐demographic backgrounds. These groups shaped the development of the final infographics, such as the inclusion of the glossary on the reverse, colours, imagery and placement of text. The characteristics of the contributors and the feedback we received from each of the groups is outlined in a report on OSF (https://osf.io/nghyk/). A control infographic on the importance of the ocean, produced by the National Oceanic and Atmospheric Administration, was chosen as it was a similar length and used a similar colour scheme to our cervical screening infographics. Full infographics are in File S2.

**TABLE 1:** Messages used in the cervical screening infographic.

	Generic cervical screening infographic for all those eligible to be screened (25–64 years)	Age‐targeted cervical screening infographic for 50‐ to 64‐year‐olds
Social norm message	7 out of 10 people who are invited get screened	4 out of 5 people in your age group, who are invited, get screened
Outcome expectations	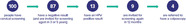	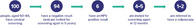
Response efficacy message	If you do not get screened, your chance of getting cervical cancer will be much higher. Screening is available every 3 years from age 25 to 49 and every 5 years from age 50 to 64	If you do not get screened, your chance of getting cervical cancer will be much higher. Screening is available every 5 years from age 50 to 64
Risk reduction message	If you are screened when you are due, you are much less likely to get cervical cancer 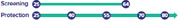	Screening every 5 years between 50 and 64 provides protection against cervical cancer lasting into your 80s 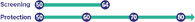
Support + discomfort acknowledgement	If you are worried about screening talk to your nurse or doctor *‘It's really important to attend cervical screening, so everyone should look out for their invitation. Early treatment of abnormal cells can prevent cancer so it's well worth it. If you're worried about discomfort, ask for information or advice about this when you book your appointment’*. Dr Nicola Weaver, GP	Screening can be more uncomfortable after menopause, but the nurse can suggest ways to make it easier, like prescribing oestrogen cream *‘It's really important to carry on attending for cervical screening after you turn 50, so everyone should look out for their invitation. Early treatment of abnormal cells can prevent cancer so it's well worth it. If you're worried about discomfort, ask for information or advice about this when you book your appointment’*. Dr Nicola Weaver, GP
Timeline	HPV can take 10–30 years to develop into cancer. You could still be at risk if you have only had one partner or not been sexually active for a long time	

### Measures

Full measures are presented in the protocol (https://osf.io/6fbrx).

#### Intention strength

Intention strength was assessed prior to randomization and again after exposure to the infographic. Intention strength was measured using three items adapted from previous studies (Cooke & Sheeran, 2013; Sheeran et al., 2017; Sheeran & Orbell, 2000): ‘I intend to go for cervical screening when I am next invited’, ‘I will try to go for cervical screening when I am next invited’ and ‘I am going to go for cervical screening when I am next invited’ (using a 7‐point Likert scale, from *strongly disagree* to *strongly agree*). These items were used to create a mean intention strength score (giving a range of 1–7). Internal reliability was very high for the three intention items at both time points (Cronbach's alpha = .94–95).

#### Knowledge and attitudes

Knowledge and attitudes related to cervical screening were assessed after exposure to the infographic. Questions were related to social norms, peace of mind, fear of result, experiential risk, affective risk, timeline of cancer development and discomfort during screening (Cervical CAM Toolkit, 2011; Ferrer et al., 2016; Hill et al., 2021; Macedo et al., 2012; Marlow et al., 2018; von Wagner et al., 2019). A mean score for beliefs about timeline was created using the two items as the statements were similar (‘HPV only takes a short time to develop into cervical cancer’/‘I believe an HPV infection can develop into cervical cancer very quickly’) and highly correlated, r(1807) = 0.84, p < .001.

#### Engagement

Engagement was measured for older women after the infographic was presented, using six bipolar adjectives adapted from a previous study (Comello et al., 2016). Participants were asked how engaging or unengaging, unappealing or appealing, unpleasant or pleasant, boring or interesting, hard or easy to understand, uninformative or informative they found the infographic using a semantic differential scale from 1 to 5. A total mean engagement score was created from the six engagement items (Cronbach's alpha = .88). We also asked all participants how much of the infographic they had read on a scale from 1 (*none of it*) to 5 (*all of it*), re‐coded into ‘less than all of it’ (1–4) versus ‘all of it’ (5).

#### Participant characteristics

Demographic information was collected before exposure to the infographic. Participants were asked their age, marital status, work status, ethnicity, education and region of residence.

Screening history was assessed by asking when participants had been for their last cervical screening test (Macedo et al., 2012; Marlow et al., 2020), re‐coded as ‘up‐to‐date’, ‘overdue’ and ‘never attended’.

Numeracy was assessed using the three‐item version of the Subjective Numeracy Scale (SNS‐3; McNaughton et al., 2015). Numeracy was assessed as it might influence interpretation of numerical health information (Reyna & Brainerd, 2008).

### Sample size

We needed 360 participants for each exposure‐by‐age group to detect a small between‐group difference in intention to attend screening (*f* = .15, based on a difference of .3 between means) with 80% power and alpha = .05. For the binary attitude items, 360 per group allowed us to estimate a 10% difference between groups with 80% power. This assumes alpha = .01 to allow for multiple comparisons. Therefore, we needed 720 women aged 25–64 years for Hypotheses 1 and 2 and 720 women aged 50–64 years for Hypotheses 3 and 4. To keep the numbers in each age‐by‐exposure group the same, we aimed for a sample of 360 women in each group, with an overall sample size of 1800 (made up of two exposure groups for 25‐ to 49‐year‐olds and three groups for 50‐ to 64‐year‐olds), as outlined in Table 1 of the protocol (https://osf.io/6fbrx). Within this sample, quotas were set for age, ethnicity and education, as specified in the protocol. Ethnicity and education quotas were set so the sample would be broadly representative of the wider population (we aimed to recruit 15% of participants from an ethnic minority background and 70% of participants with education below degree level). However, the education and ethnicity quotas were removed for 50‐ to 64‐year‐olds towards the end of data collection as uptake was low in some groups and only age quotas were specified.

### Data analysis

All analyses were carried out in SPSS version 27.0 using pre‐written syntax. To assess the effect of the infographics on intention, comparisons were performed between the following: (i) generic and control infographic groups for 25‐ to 64‐year‐olds and (ii) generic and age‐targeted infographic groups for 50‐ to 64‐year‐olds. Post‐exposure mean intention was negatively skewed due to a high proportion of participants with the highest intention score (skewness = −1.62; kurtosis = 1.51). Despite this, we ran ANCOVAs to enable a comparison with previous research (Marlow et al., 2021; Waite et al., 2022), as there is evidence that ANCOVAs can be run with skewed data in randomized trials (Vickers, 2005). The ANCOVAs were conducted with intention as the dependent variable and baseline intention and numeracy as covariates. We also ran sub‐group analyses with two groups: (i) a pre‐specified analysis with only participants who reported reading all the infographic (as a ‘per protocol’ analysis) and (ii) an exploratory analysis with participants whose intention score was lower than the maximum (7) at baseline (to avoid a ceiling effect).

Logistic regression models were conducted with knowledge and attitude items as the dependent variables (recoded as dichotomous outcomes, as pre‐specified in the protocol https://osf.io/6fbrx). To assess the effect of age targeting on engagement in older women, an ANCOVA was run with engagement as the dependent variable and age, baseline intention and numeracy as covariates. The engagement variable was moderately negatively skewed (skewness = −.63; kurtosis = .03).

## RESULTS

**FIGURE 1:**
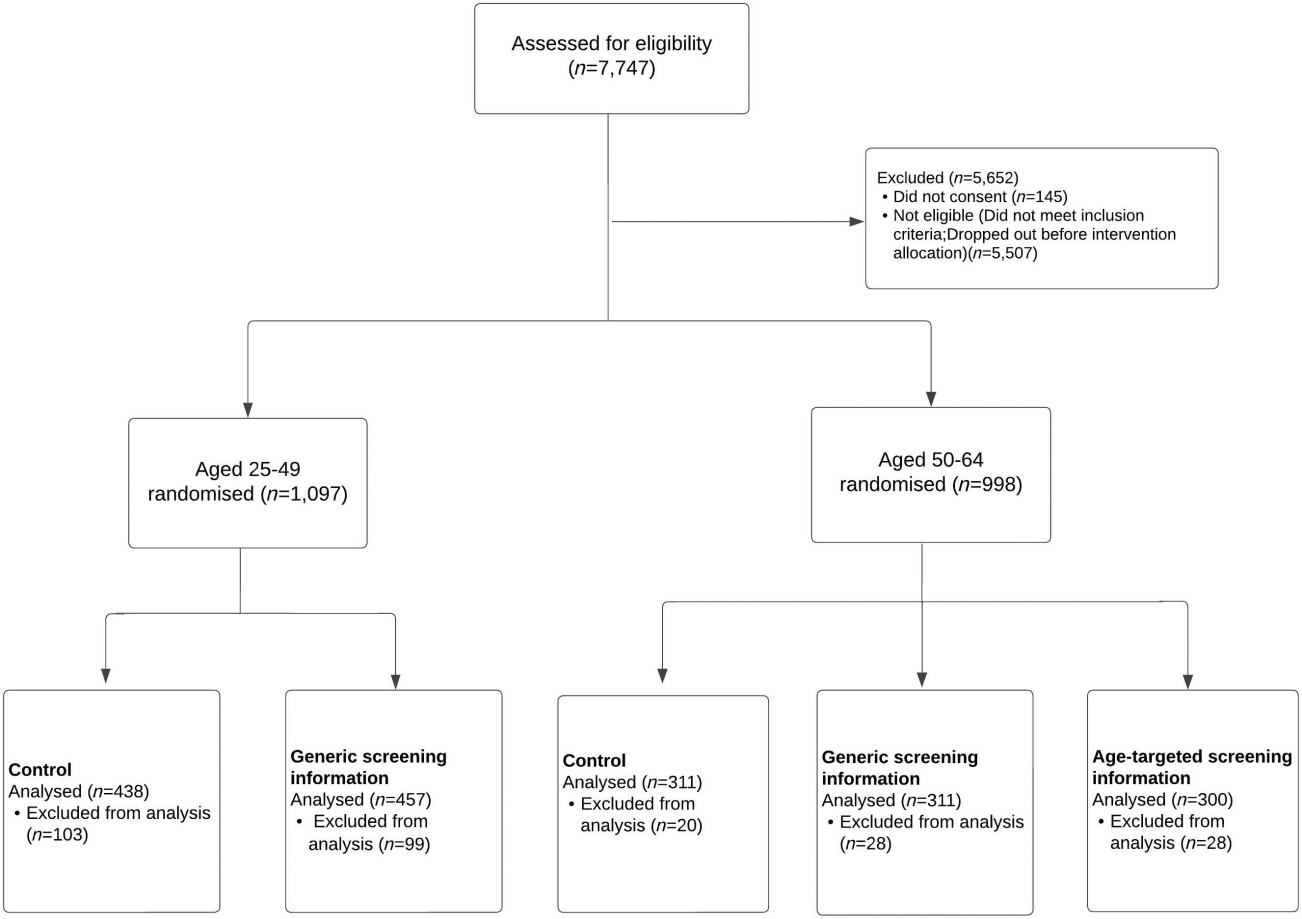
Participant flow diagram. *Note*: Participants were excluded from the analysis if they failed the attention check, were too slow or took too long completing the survey, or dropped out before completing their post‐intention.

Of the 7747 women who followed the link to the survey, 2095 consented, were eligible and were allocated to an intervention group. Participants were excluded (*n* = 278) if they completed the survey too quickly (under 2 min) or too slowly (over 3.5 hr), failed the attention check or dropped out before reporting their post‐exposure intention. Therefore, there were 1817 women whose data were included in the analyses (25–49 years = 895; 50–64 years = 970; see Figure 1 for numbers in each exposure group).

### Sample characteristics

Sample characteristics are reported in Table 2. Participants had an average age of 46.7 and most were up to date with cervical screening (68.8%). We used quotas for ethnicity and education. In the final sample, 10.4% of participants were from an ethnic minority background and 66.3% did not have a degree level qualification or above.

**TABLE 2:** Sample characteristics (*n* = 1817) showing randomization across the two age groups.^a^

	Overall (*n* = 1817)	25‐ to 49‐year‐olds		50‐ to 64‐year‐olds		
		Control (*n* = 438)	Generic screening information (*n* = 457)	Control (*n* = 311)	Generic screening information (*n* = 311)	Age‐targeted screening information (*n* = 300)
Age (years), mean (*SD*)	46.68 (11.80)	36.86 (7.25)	35.87 (7.47)	56.23 (4.02)	56.80 (4.16)	57.12 (4.33)
Age group, *n* (%)						
25–35	445 (24.5)	200 (45.7)	245 (53.6)	–	–	–
36–49	450 (24.8)	238 (54.3)	212 (46.4)	–	–	–
50–64	922 (50.7)	–	–	311 (100.0)	311 (100.0)	300 (100.0)
Education, *n* (%)						
Low‐level	29 (1.6)	6 (1.4)	10 (2.2)	4 (1.3)	2 (.6)	7 (2.3)
Mid‐level	1167 (64.2)	284 (64.8)	283 (61.9)	198 (63.7)	201 (64.6)	201 (67.0)
High‐level	612 (33.7)	145 (33.1)	162 (35.4)	107 (34.4)	108 (34.7)	90 (30.0)
Other	2 (.1)	0 (.0)	0 (.0)	1 (.3)	0 (.0)	1 (.3)
Prefer not to say	7 (.4)	3 (.7)	2 (.4)	1 (.3)	0 (.0)	1 (.3)
Marital status, *n* (%)						
Single	468 (25.8)	152 (34.7)	163 (35.7)	56 (18.0)	47 (15.1)	50 (16.7)
Married/civil partnership/co‐habiting	1111 (61.1)	261 (59.6)	268 (58.6)	189 (60.8)	200 (64.3)	193 (64.3)
Separated/divorced/widowed	231 (12.7)	24 (5.5)	23 (5.0)	65 (20.9)	63 (20.3)	56 (18.7)
Prefer not to say	7 (.4)	1 (.2)	3 (.7)	1 (.3)	1 (.3)	1 (.3)
Work status, *n* (%)						
Employed	1214 (66.8)	325 (74.2)	351 (76.8)	196 (63.0)	190 (61.1)	152 (50.7)
Not working	581 (32.0)	104 (23.7)	97 (21.2)	113 (36.6)	120 (38.6)	147 (49.0)
Student	14 (.8)	6 (1.4)	6 (1.3)	1 (.3)	0 (.0)	1 (.3)
Prefer not to say	8 (.4)	3 (.7)	3 (.7)	1 (.3)	1 (.3)	0 (.0)
Ethnic background, *n* (%)						
Any White	1628 (89.6)	378 (86.3)	401 (87.7)	284 (91.3)	282 (90.7)	283 (94.3)
Mixed ethnic background	42 (2.3)	17 (3.9)	15 (3.3)	8 (2.6)	2 (.6)	0 (.0)
Any Asian background	81 (4.5)	25 (5.7)	24 (5.3)	10 (3.2)	13 (4.2)	9 (3.0)
Any Black background	47 (2.6)	12 (2.7)	10 (2.2)	8 (2.6)	11 (3.5)	6 (2.0)
Other	15 (.8)	6 (1.4)	5 (1.1)	0 (.0)	2 (.6)	2 (.7)
Prefer not to say	4 (.2)	0 (.0)	2 (.4)	1 (.3)	1 (.3)	0 (.0)
Screening status, *n* (%)						
Up to date	1251 (68.8)	280 (63.9)	285 (62.4)	208 (66.9)	240 (77.2)	238 (79.3)
Overdue	391 (21.5)	92 (21.0)	104 (22.8)	84 (27.0)	62 (19.9)	49 (16.3)
Never attended	175 (9.6)	66 (15.1)	68 (14.9)	19 (6.1)	9 (2.9)	13 (4.3)
Baseline screening intention (range 1–7), mean (*SD*)	5.79 (1.74)	5.80 (1.78)	5.88 (1.63)	5.65 (1.84)	5.77 (1.75)	5.82 (1.75)

^a^ Analyses addressing Hypotheses 1 and 2 were with all women aged 25–64 years and compared the two control groups combined (total *n* = 749) with the two ‘generic screening information’ groups combined (total *n* = 756). Analyses addressing Hypotheses 3, 4, 5 were with women aged 50–64 years only and compared ‘generic screening information’ (*n* = 311) with ‘age‐targeted screening information’ (*n* = 300).

### The impact of a cervical screening infographic for all screening age women

Hypothesis 1 In line with our first hypothesis, among all screening age women (25–64 years), those who were shown the screening infographic had significantly higher mean intention scores than those who were shown the control infographic [*F*(1, 1513) = 6.14, *p* = .013, $\eta_{p}^{2}$ = .004], but the absolute difference was small (5.91 vs. 5.84 out of a possible 7).

Hypothesis 2 Those who were shown an infographic about cervical screening had better knowledge of the timeline for cervical cancer development (OR: 5.18, 95% CI: 3.86–6.95) and were more likely to correctly judge how many women go for cervical screening (OR: 3.03, 95% CI: 2.38–3.87). Seeing the cervical screening infographic also resulted in more positive beliefs about the benefits of screening (OR: 2.23, 95% CI: 1.52–3.28) compared to the control group. There were no significant differences between the two groups for any other knowledge or attitude measures (see Table 3).

**TABLE 3:** Knowledge and attitudes in women aged 25–64 years (*n* = 1517).

	Control (*n* = 749)	Generic screening information (*n* = 768)	Unadjusted odds ratio relative to the control group (95% CI)
	*n* (%)	*n* (%)	
Descriptive social norms for Cx screening			
Incorrect	626 (83.8)	481 (63.0)	–
Correct	121 (16.2)	282 (37.0)	3.03 (2.38–3.87)
Cx screening important for peace of mind			
Disagree/neutral	116 (15.5)	116 (15.2)	–
Agree	631 (84.5)	647 (84.8)	1.03 (.78–1.36)
Scared of what Cx screening might find			
Disagree/neutral	338 (45.2)	359 (47.1)	–
Agree	409 (54.8)	404 (52.9)	.93 (.76–1.14)
Feel vulnerable to cervical cancer			
Disagree/neutral	571 (76.4)	587 (76.9)	–
Agree	176 (23.6)	176 (23.1)	.97 (.77–1.24)
Worry about developing cervical cancer			
Not at all/slightly	244 (32.7)	271 (35.5)	–
Somewhat/moderately/extremely	503 (67.3)	492 (64.5)	.88 (.71–1.09)
Screening reduces CaCx risk			
Disagree/neutral	86 (11.5)	42 (5.5)	–
Agree	661 (88.5)	721 (94.5)	2.23 (1.52–3.28)
Long timeline from HPV to CaCx			
Incorrect/do not know	681 (91.2)	508 (66.6)	–
Correct	66 (8.8)	255 (33.4)	5.18 (3.86–6.95)
Screening can be made less uncomfortable			
Disagree/ neutral	62 (8.3)	54 (7.1)	–
Agree	685 (91.7)	709 (92.9)	1.19 (.81–1.74)

*Note*: Disagree = ‘disagree’/‘strongly disagree’; Agree = ‘agree’/‘strongly agree’; neutral = ‘neither agree nor disagree’. Social norms coded as correct when 7 in every 10 were selected. Timeline was correct if participants had a mean score >3, which corresponded to strongly disagree or disagree. Numbers may differ due to missing data.

Abbreviations: CaCx, cervical cancer; Cx, cervical.

#### Sub‐group analyses

##### Pre‐specified

The effect of the screening infographic on intention was slightly greater in the sub‐group who reported reading the whole infographic; mean intention scores were 6.09 versus 5.98 for the intervention and control groups respectively [*F*(1, 730) = 8.34, *p* = .004, $\eta_{p}^{2}$ = .011].

##### Exploratory

The effect of the screening infographic on intention was similar when we excluded participants with the highest possible intention at baseline [mean post‐exposure intention was 4.70 vs. 4.83; *F*(1, 754) = 6.21, *p* = .013, $\eta_{p}^{2}$ = .008].

### The impact of an age‐targeted infographic for older women

Hypothesis 3 Contrary to our hypothesis, among older women (50–64 years), there was no significant difference in mean intention scores between those shown the age‐targeted screening infographic and those shown the generic screening infographic [5.90 vs. 5.89; *F*(1, 607) = .03, *p* = .853, $\eta_{p}^{2}$ < .001].

Hypothesis 4 Women aged 50–64 years who were shown the age‐targeted infographic had lower odds of getting the social norms question correct (i.e., accurately recalling how many women they were told go for cervical screening) than those shown the generic infographic (OR: .50, 95% CI: .35–.71), which was contrary to our hypothesis. There were no differences between the two groups for the other knowledge and attitude measure (see Table 4).

Hypothesis 5 Mean scores and the standard deviation for each item assessing engagement are presented in Table 4. Among older women, those who were shown the age‐targeted infographic had a small but significantly higher engagement score than those shown the generic version [4.00 (*SD* = .95) vs. 3.80 (*SD* = 1.10) out of a possible 5; *F*(1, 608) =9.48, *p* = .002, $\eta_{p}^{2}$ = .015].

**TABLE 4:** Knowledge and attitudes in women aged 50–64 years (*n* = 611).

	Generic screening information (*n* = 311)	Targeted screening information (*n* = 300)	Unadjusted odds ratio relative to the generic group (95% CI)
	*n* (%) or mean (*SD*)	*n* (%) or mean (*SD*)	
Descriptive social norms for Cx screening			
Incorrect	188 (60.6)	226 (75.6)	–
Correct	122 (39.4)	73 (24.4)	.50 (.35–.71)
Cx screening important for peace of mind			
Disagree/neutral	54 (17.4)	38 (12.7)	–
Agree	256 (82.6)	261 (87.3)	1.50 (.92–2.27)
Scared of what Cx screening might find			
Disagree/neutral	168 (54.2)	180 (60.2)	–
Agree	142 (45.8)	119 (39.8)	.78 (.57–1.08)
Feel vulnerable to cervical cancer			
Disagree/neutral	269 (86.8)	248 (82.9)	–
Agree	41 (13.2)	51 (17.1)	1.35 (.86–2.11)
Worry about developing cervical cancer			
Not at all/slightly	139 (44.8)	145 (48.5)	–
Somewhat/moderately/extremely	171 (55.2)	154 (51.5)	.86 (.63–1.19)
Screening reduces CaCx risk			
Disagree/neutral	19 (6.1)	14 (4.7)	–
Agree	291 (93.9)	285 (95.3)	1.33 (.65–2.70)
Long timeline from HPV to CaCx			
Incorrect/do not know	199 (64.2)	203 (67.9)	–
Correct	111 (35.8)	96 (32.1)	.85 (.61–1.19)
Screening can be made less uncomfortable			
Disagree/neutral	28 (9.0)	15 (5.0)	–
Agree	282 (91.0)	284 (95.0)	1.88 (.98–3.60)
Amount of infographic read			
Read all of it	173 (55.6)	182 (60.7)	–
Did not read all of it	138 (44.4)	118 (39.3)	–
Individual items assessing engagement (range 1–5), mean (*SD*)			
Appealing	3.53 (1.06)	3.73 (1.00)	–
Pleasant	3.45 (.95)	3.62 (.94)	–
Interesting	3.62 (1.12)	3.88 (1.00)	–
Easy to understand	4.25 (.88)	4.42 (.81)	–
Informative	4.29 (.82)	4.39 (.78)	–

*Note*: Disagree = ‘disagree’/‘strongly disagree’; Agree = ‘agree’/‘strongly agree’; neutral = ‘neither agree nor disagree’. Social norms coded as correct when 4 in every 5 were selected. Timeline was correct if participants had a mean score >3, which corresponded to strongly disagree or disagree. Numbers may differ due to missing data.

Abbreviations: CaCx, cervical cancer; Cx, cervical.

#### Sub‐group analyses

##### Pre‐specified

There was no significant difference in mean intention scores between those shown the age‐targeted screening infographic and those shown the generic screening infographic when we repeated the analysis with the sub‐group who reported reading the whole infographic [6.12 vs. 6.08; *F*(1, 351) =.77, *p* = .382, $\eta_{p}^{2}$ = .002].

##### Exploratory

There was no significant difference in mean intention scores between those shown the age‐targeted screening infographic and those shown the generic screening infographic when we excluded those with the highest possible intention at baseline [4.77 vs. 4.72; *F*(1, 293) = .46, *p* = .498, $\eta_{p}^{2}$ = .002].

## DISCUSSION

This study investigated the effect of generic and age‐targeted cervical screening infographics on intention, knowledge, attitudes and engagement. Viewing a cervical screening infographic resulted in a higher intention to be screened across all screening age women compared to a control infographic, but the difference was small. For older women, an age‐targeted infographic had the same impact as the generic infographic. Excluding those with the highest possible intention score at baseline or those who reported not reading all the infographic did not make a difference to these findings. Across all screening age women, the cervical screening infographic resulted in more positive attitudes and knowledge of how many people go for cervical screening, the benefits of screening and more accurate beliefs about the timeline of cervical cancer development. Targeted information for older women did not change attitudes or knowledge any more than the generic leaflet. However, the targeted information did result in greater engagement than the generic version.

A very small difference in intention was found between participants who viewed the generic infographic and the control infographic. This supports findings exploring the use of printed text‐based educational interventions for increasing screening uptake. For example, a pamphlet including basic knowledge about cervical screening was found not to increase uptake compared to a control group (Bowman et al., 1995). There is also evidence that while an invitation letter increases cervical screening uptake, using an information brochure alongside it does not increase attendance further (Acera et al., 2017; Eaker et al., 2004; Radde et al., 2016). The very small difference found in our study, and not in previous research, could be due to the fact we measured intention rather than screening uptake, as there is likely to be a reduction in the number of people who go for screening compared to those who intend to go (Ajzen, 2011). It could also be due to the use of theory informing the messages, or the format of an infographic being more engaging compared to an information brochure. There is evidence of other interventions using printed educational materials being effective, but these have used tailored materials and included other intervention components such as invitation letters (Decker et al., 2013; McAvoy & Raza, 1991; Rimer et al., 1999).

Previous work (Marlow et al., 2021; Waite et al., 2022) suggested that reading a set of age‐targeted messages might improve intention in older women, but this did not seem to be the case in this study. In line with the findings of this study, research evaluating an age‐targeted invitation letter for older women did not find an increase in cervical screening attendance compared to a generic one (Mullins, 2009). Targeting is most effective at changing behaviour when there are no large variabilities within the target group that might affect the target behaviour (Kreuter & Wray, 2003). However, there is evidence to suggest that socio‐demographic variables such as ethnicity and deprivation are related to intention to attend cervical screening (Wilding et al., 2022), which may have reduced the effectiveness of an infographic targeted solely by age.

We found that there was greater knowledge and more positive attitudes towards cervical screening among women aged 25–64 years who were shown cervical screening information than those viewing an unrelated infographic. Previous studies have also found that educational interventions can improve attitudes and knowledge of cervical cancer. To our knowledge, most studies using printed educational resources to increase uptake have not included measures of attitudes and knowledge. However, studies evaluating educational interventions such as health talks and workshops have been shown to improve knowledge and attitudes (Jandorf et al., 2008; Mishra et al., 2009; O'Brien et al., 2010; Rosser et al., 2015; Thompson et al., 2017). These studies used more intensive interventions than this study and cultural tailoring. It is encouraging that a generic infographic can have a positive effect on attitudes and knowledge, as it can be delivered at a lower cost and is easily scalable.

The age‐targeted infographic had no greater effect on attitudes and knowledge than the generic infographic. These findings are similar to those of previous studies which found age‐targeted messages did not result in more positive attitudes and better knowledge than generic messages (Marlow et al., 2021; Waite et al., 2022). However, the targeted information was found to be more engaging than generic information in this study. This supports the hypothesis that targeted information is likely to be more actively and carefully processed if the information presented can be linked to personal experience (Hawkins et al., 2008; Kreuter & Wray, 2003).

There were both strengths and limitations of this study. Using an online stratified RCT design allowed us to efficiently compare two intervention groups in the same study. However, using an online panel for recruitment may have increased self‐selection bias, and we were unable to meet the quotas we set for education and ethnicity. However, we still had a high number of participants from an ethnic minority and without a degree take part in the study. Additionally, only including participants who had access to a computer or tablet meant that those without the technology to access the survey were excluded, reducing the generalizability of the findings. Finally, there is often a gap between intention and action (Ajzen, 2011). As we only measured intention and not behaviour, we were not able to assess whether the infographic helped to move participants who had the highest intentions at baseline into action.

Despite these limitations, this study suggests that infographics could be used to increase intention to attend cervical screening. While there was only a small difference in intention, the small effect size found in this study could have a big impact at a population level. Additionally, it is likely that multiple strategies are required to result in an increase in uptake across the population in England. Infographics could be used primarily to increase screening related knowledge, supporting informed choice. Due to the higher level of engagement in the age‐targeted infographic, targeted information could also be used to inform older women of changes to the screening programme for example changes to screening intervals. As the age‐targeted information had no effect on intention, future research could look at targeting information to multiple characteristics, such as age and deprivation, rather than solely targeting based on one characteristic to see if this increases intention. Additionally, the majority of participants in this study were up to date with screening, and it is possible that the infographic would have a larger effect in a sample who are currently overdue for screening.

## CONCLUSION

This study aimed to test the effect of two cervical screening infographics, one generic and one age‐targeted, on intention, attitudes, knowledge and engagement. We found that in the context of cervical screening, when women are likely to have already been sent information on screening, an infographic can result in more positive attitudes and better knowledge but is unlikely to have a large impact on screening behaviour. Additionally, age‐targeted information does not shift attitudes, knowledge and intention further than generic information in older women, but may have a positive impact on engagement and therefore encourage women to read and process materials.

## AUTHOR CONTRIBUTIONS

**Frances Waite:** Formal analysis; investigation; writing – original draft; project administration. **Laura A.V. Marlow:** Conceptualization; methodology; formal analysis; writing – review and editing. **Martin Nemec:** Conceptualization; writing – review and editing. **Jo Waller:** Conceptualization; methodology; writing – review and editing; supervision; funding acquisition.

## CONFLICT OF INTEREST STATEMENT

The authors declare that they have no known competing financial interests or personal relationships that could have appeared to influence the work reported in this article.

## Supporting information


File S1.

File S2.

## Data Availability

The data that support the findings of this study are openly available on Open Science Framework at https://osf.io/rv5qn.
