# Supplementary material for: The impact of age‐relevant and generic infographics on knowledge, attitudes and intention to attend cervical screening: A randomized controlled trial
Source: Br J Health Psychol. 2023 Sep 28;29(1):204–20. doi: 10.1111/bjhp.12695 (PMC10952565; doi:10.1111/bjhp.12695)
Supplement: Supplementary file 2 — File S2. [file BJHP-29-204-s001.docx]

**Supplementary File 2: Infographics**

**Generic infographic**


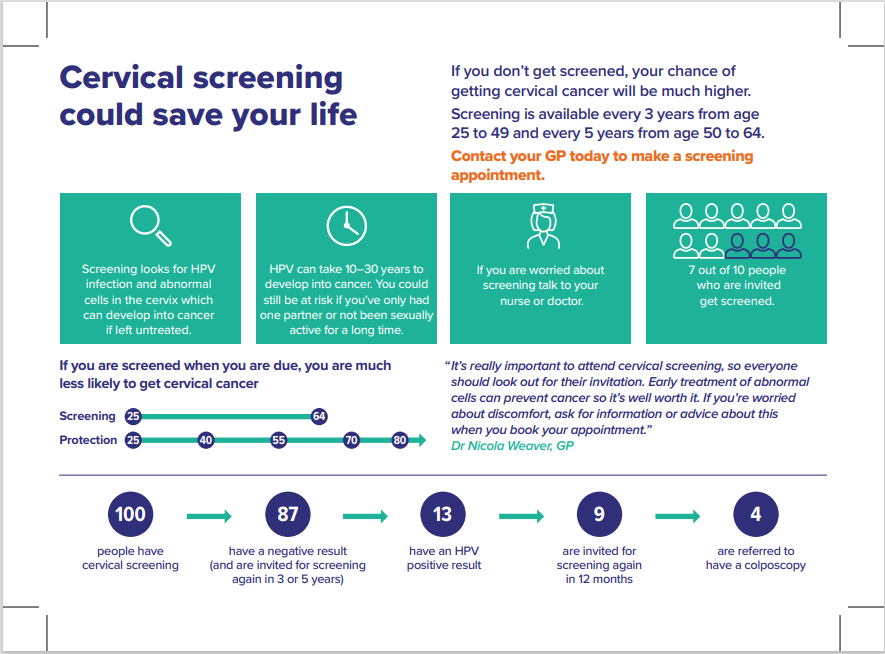


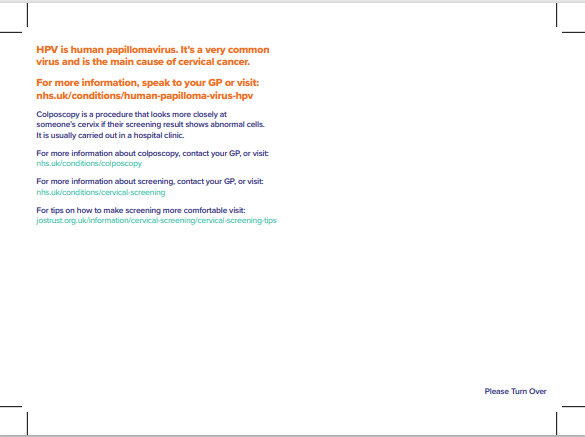


**Age-targeted infographic**


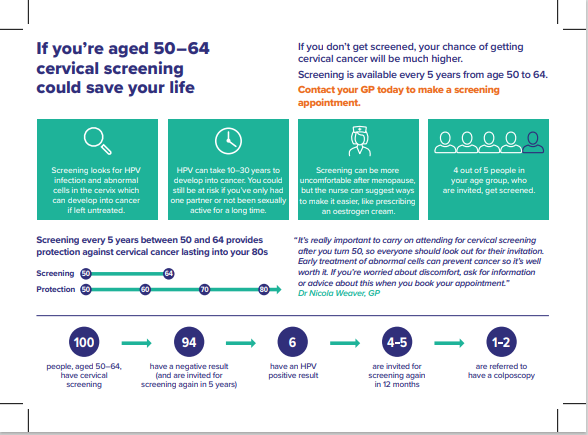


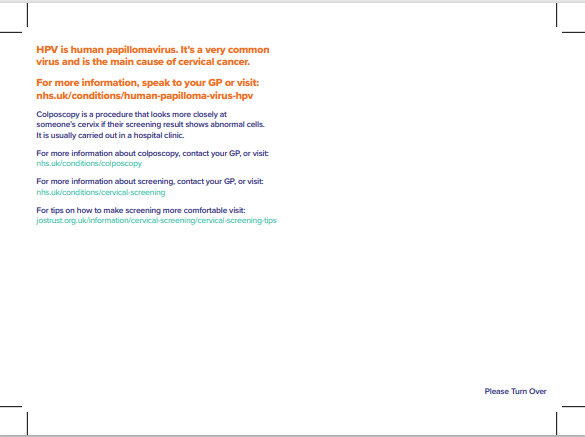


**Control infographic**


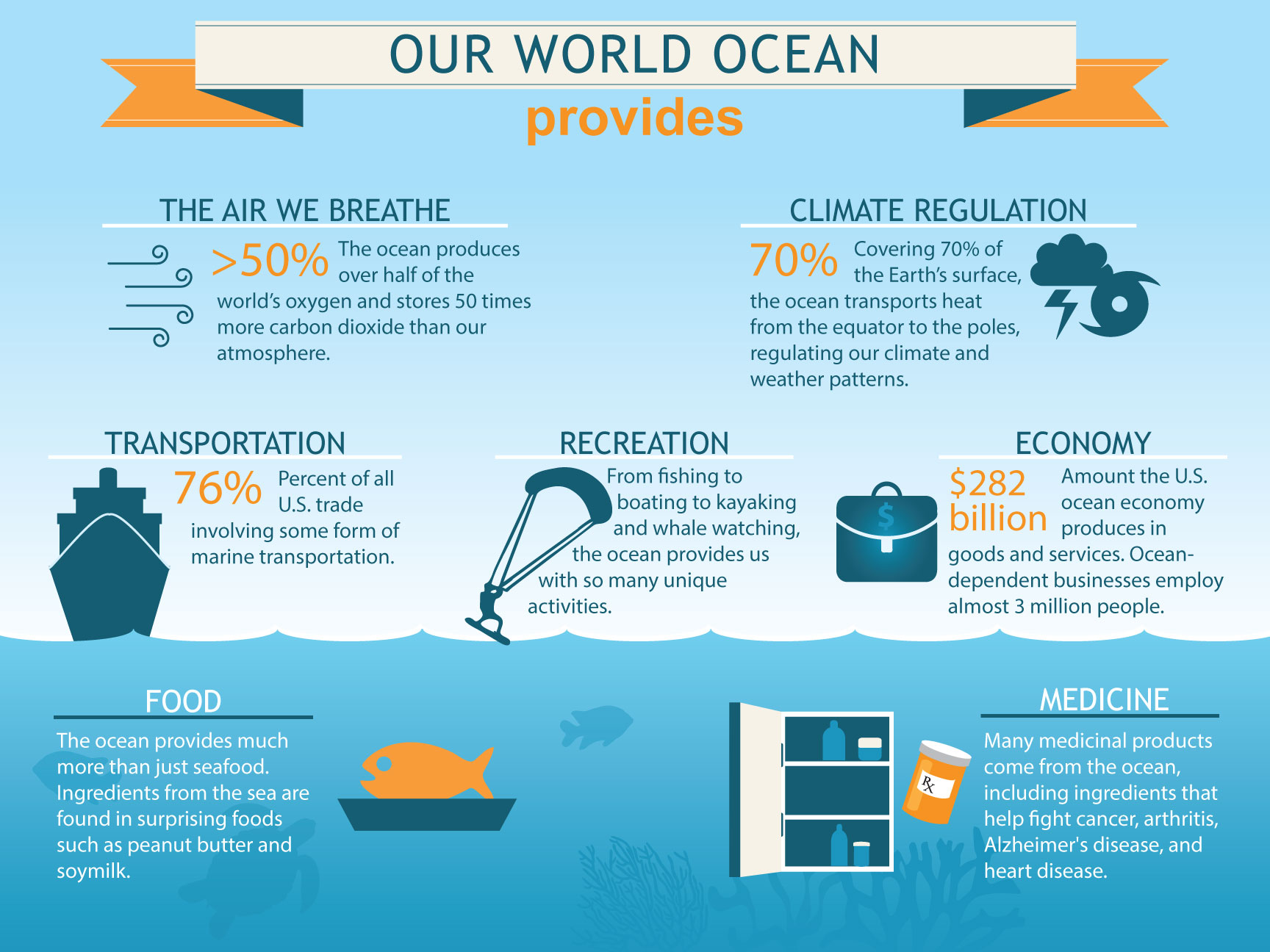


*National Oceanic and Atmospheric Administration. Why should we care about the ocean? Available from: https://oceanservice.noaa.gov/facts/why-care-about-ocean.html*
